# A combined model of child malnutrition and morbidity in Ethiopia using structural equation models

**DOI:** 10.1038/s41598-023-27440-7

**Published:** 2023-01-10

**Authors:** Kasahun Takele, Temesgen Zewotir, Denis Ndanguza

**Affiliations:** 1 African Center of Excellence in Data Science, University of Rwanda, Kigali, Rwanda; 2 School of Mathematics, Statistics and Computer Sciences, University of KwaZulu-Natal, Durban, South Africa; 3 College of Science and Technology, University of Rwanda, Kigali, Rwanda

**Keywords:** Diseases, Medical research, Risk factors

## Abstract

Malnutrition and morbidity are substantial problems in Ethiopia and are still pervasive and persistent. Despite this, there has been scant research on the coexistence of malnutrition and morbidity indicators. Moreover, previous studies were based on all data records of measurements from manifest data. Thus, this study aims to identify the correlates and coexistence of child malnutrition and morbidity within this country. Cross-sectional data which is collected by Ethiopia Demographic and Health Survey were used. The generalized structural equation models were used to examine the association between child malnutrition, morbidity, and potential risk factors. The generalized structural equation models help to provide latent effects of child malnutrition and morbidity within a combined modeling framework. In addition, the generalized structural equation models make it possible to analyze malnutrition as a mediator of the association between selected risk factors and latent variable morbidity. The data analysis was done using SPSS AMOS and R software. The analysis indicated that children born to nourished mothers (AOR = 0.71, 95% CI 0.68–0.75), born to enough birth space between 24 and 47 months and (AOR = 0.93, 95% CI 0.88–0.99), 48 months and above (AOR = 0.71, 95% CI 0.65–0.76), being from middle-income households (AOR = 0.85, 95% CI 0.78–0.91), high-income households (AOR = 0.66, 95% CI 0.61–0.72), from mother with primary or secondary (AOR = 0.79, 95% CI 0.75–0.85) and higher education level (AOR = 0.57, 95% CI 0.41–0.78) were less affected by malnutrition. It also revealed that a child born second to third (AOR = 0.87, 95% CI 0.77–0.99), fourth and higher (AOR = 0.88, 95% CI 0.79–0.99) and children from a husband-educated higher level (AOR = 0.76, 95% CI 0.64–0.89) were less likely to be ill. Children who breastfeed (AOR = 0.98, 95% CI 0.80–0.99), from nourished mothers (AOR = 0.96, 95% CI 0.94–0.097), from middle income (AOR = 0.97, 95% CI 0.96–0.99), high-income households (AOR = 0.94, 95% CI 0.93–0.96), birth spacing 24–47 months (AOR = 0.99, 95% CI 0.98–1.00) and 48 months and above (AOR = 0.96, 95% CI 0.94–0.97) were indirectly affected by morbidity via malnutrition. This investigation has revealed that childhood malnutrition and morbidity remain major child health challenges in Ethiopia with demographic, socioeconomic, maternal, child, and geographic variables playing significant roles. Efforts to resolve these issues need to take these factors into account. Therefore, malnutrition and morbidity prevention should include encouraging birth spacing, mother education programs, and breastfeeding practices.

## Introduction

The burden of child malnutrition and morbidity in low-income countries is devastating, and these situations commonly coexist. They are among the serious health problems in less developed countries. Malnutrition arises from insufficient intake or an inappropriate proportion of diets. Excess nutrient losses or use results in malnutrition. Undernutrition arises due to an insufficient intake of nutritional energy or other nutrients. Morbidity is a departure from health. Morbidity in children could be in terms of infectious diseases such as diarrhea, fever, cough, pneumonia, and tetanus, or chronic such as congenital anomalies and thalassemia. Childhood undernutrition is directly associated with child morbidity and mortality because it compromises the immune function; exacerbates vulnerability to infectious diseases, and speeds up the progression, severity, and period of the disease. The disease is a cause and consequence of undernutrition. Understanding the factors that devastate the nutritional and health status of under-five children in developing countries, particularly Ethiopia is a vigorous research problem. In general, even the world pays attention to understanding the relationship between infection and nutrition. Addressing only child diseases is questionable to be successful in reducing the prevalence of child malnutrition^1^. Investigating the impact of important risk factors on child morbidity and malnutrition is of high relevance for developing countries. However, previous researchers have usually carried out separate regression analyses for certain diseases or types of malnutrition, neglecting possible associations between them^2–4^. The massive burden of child death results from undernutrition, as a result of its potentiating effect on common infectious due to the high prevalence of infectious diseases in developing countries, contributes significantly to malnutrition. Likewise, malnutrition increases one’s vulnerability to infections and is thus a major component of illness and death from disease. Undernourishment is subsequently the most significant risk factor for the burden of disease in developing countries^2,6^. It has become clear that whilst malnutrition results in increased incidence, severity, and case fatality of common infections, risks continue beyond acute episodes resulting in significant post-discharge mortality. A well-established concept of a vicious cycle between nutrition and infection has now evolved to encompass dysbiosis and pathogen colonization as precursors to infection; enteric dysfunction constituting malabsorption, dysregulation of nutrients and metabolism, inflammation, and bacterial translocation. All of these interact with a child’s diet and environment^7^.

Global studies indicated that the increased vulnerability happens across the nutritional status range. The increased childhood death is associated with malnutrition and infectious diseases^8^. Furthermore, children with severe malnutrition are at high risk of death from diarrhea, pneumonia, fever, cough, and malaria. Consequently, nourishment-associated factors donate to about 45 percent, pneumonia 15 percent, diarrhea 8 percent, and malaria 5 percent of death in children under five years of age^9^. Likewise, in Ethiopia among under-five children, 38 percent were stunted, 10 percent were wasted, 24 percent were underweighted, 12 percent had diarrhea, 14 percent had a fever and 16 percent had cough episodes^10^. This fact shows that Ethiopia is suffering from child malnutrition and morbidity. Children with deprived nutritional parameters show a raised risk of death from a diversity of severe infections^11^. As stated by^12^, undernourished children have a clear excess risk of communicable morbidity and mortality. This indicates that there is a vicious cycle of child morbidity and malnutrition.

Furthermore, child malnutrition and morbidity measures are directly unobservable as a single variable. Because the response variables are latent, classical regression models cannot be used to detect the common latent risk factors. Likewise, researchers are increasingly interested in malnutrition's complex coexistence with morbidity diseases. It is not well documented how stunting, wasting, underweight, diarrhea, fever, and cough interact as well as how they interact with various risk factors. Despite this, only a limited number of studies have explored the main factors linked to child malnutrition and morbidity in Ethiopia to determine the most effective interventions. Therefore, this study aims to identify common drivers of childhood malnutrition and morbidity using generalized structural equation models.

## Data and methodology

### The data

In this study, cross-sectional data which is 2016 Ethiopian Demographic and health survey datasets were used to identify the correlates and coexistence of child malnutrition and morbidity in the country. The survey EDHS 2016 used a two-stage cluster sampling design with urban–rural (region as strata), yielding 21 sampling strata. In all, 645 clusters, consisting of 202 in urban, and 443 in rural areas, and a representative sample of 15,683 households were selected for the survey interview. Of the total 15,683 women, 5348 from urban and 10,335 from rural households completed the interview. Among the sampled households, data for 8742 children under the age of five years were used. The unit of analysis in this study was children aged less than 60 months.

#### Outcome variables

The outcome variables include stunting, wasting, underweight, fever, cough, and diarrhea. Children who suffered from each of these diseases were categorized as ‘Yes’ while those who did not suffer from any of the diseases were categorized as ‘No’.

Risk factors: The socio-economic characteristics; wealth index, water source, toilet facility, mother’s work status, and internet use, child characteristics; sex of the child, age of the child, anemia level, childbirth order number, preceding birth interval, breastfeeding and child place of delivery, mother characteristics; mothers body mass index, mother education level and mother marital status, geographic characteristics; place of residence and region, and demographic characteristics which include household members were considered.

**Figure 1:**
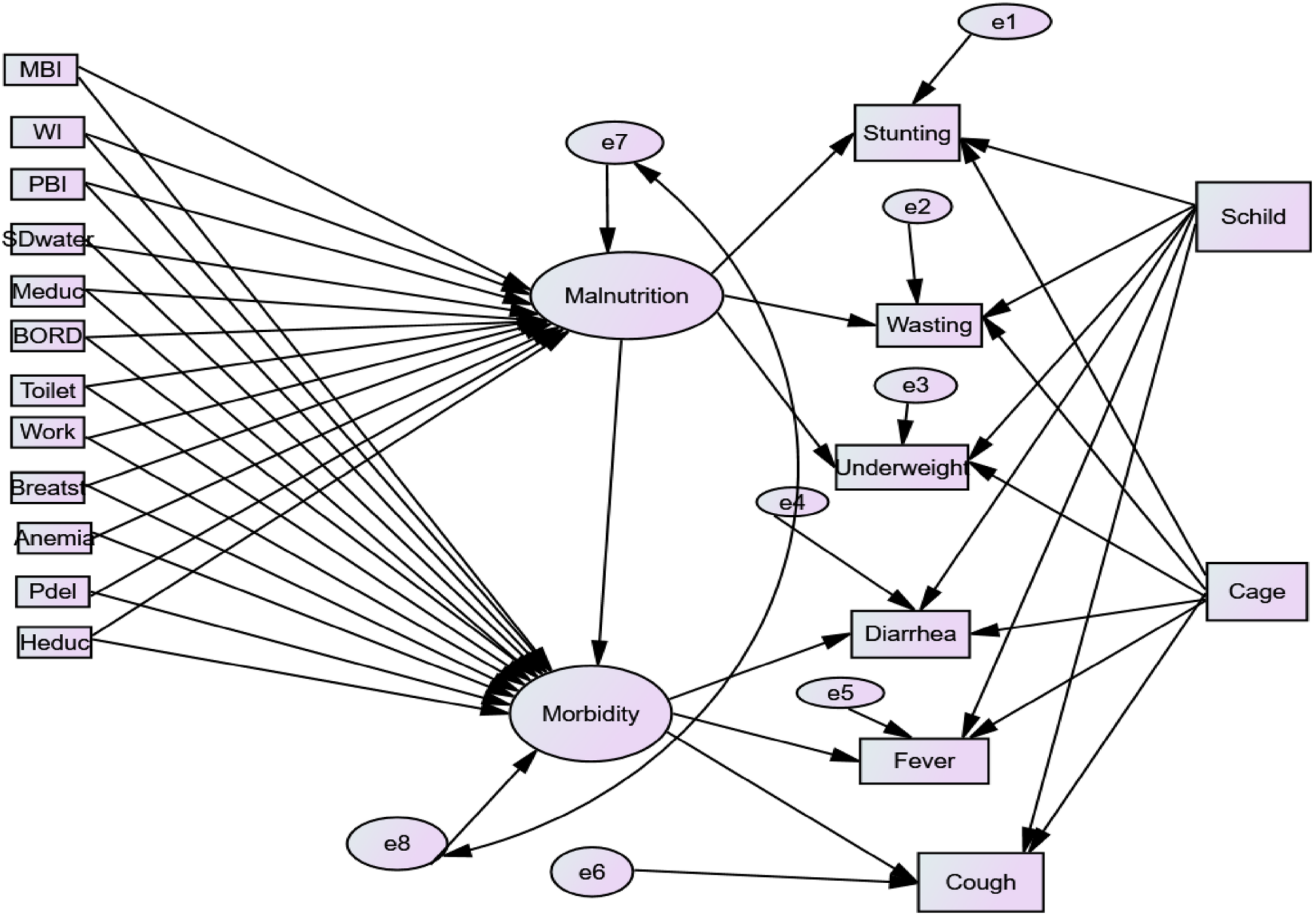
Proposed theoretical Generalized SEM path diagram of child malnutrition and morbidity in Ethiopia.

Figure 1 indicates the multiple indicators and multiple causes (MIMIC) model. In this model, malnutrition and morbidity are latent endogenous variables. We can conceptualize the six measured variables stunting, wasting, underweight, diarrhea, cough, and fever as being the realization of childhood malnutrition and morbidity, respectively. This can be quantified using the latent variables of malnutrition and morbidity. Arrows denote the direction of influence. Latent variables are indicated by circles, and manifest variables are represented by boxes. The path diagrams considered in this study are depicted hereunder.

### Generalized structural equation model

It is important to deal with unobserved (latent) variables that cannot be directly observed by a single observable variable. For instance, child malnutrition and morbidity are latent variables. The characteristics of a latent variable can be measured by a linear combination of manifest variables. The quantity of child malnutrition can be reflected by the anthropometric indices and morbidity of a child that can be measured by diarrhea, fever, and cough. Hence, we are interested in measuring the latent variables of malnutrition and morbidity via observed indicators. Then, we identify the common predictors on these latent variables, thus taking into account association within the joint model.

Structural equation modeling (SEM) is an influential multivariate technique used to examine and assess multivariate relationships. SEMs differ from other modeling approaches as they test the direct and indirect effects on pre-assumed relationships. The structural equation model can be disintegrated into two sub-models: a measurement model that designates the relationships among observed and latent variables, and a structural model that defines relationships among latent variables. Furthermore, a structural equation model specifies the way by which specific unobserved variables directly or indirectly influence/cause changes in the values of certain other unobserved variables in the model, which is maybe impossible to directly analyze using ordinary regression that is based on raw observations^12,13^. Furthermore, path diagrams are considered as a schematic presentation of structural equation models. It is used to provide a visual depiction of relations that are supposed to hold between the covariates under investigation. Path diagrams for a specific structural equation model are equivalent to its set of equations that relates dependent variables to their covariates^13^. Likewise, path diagrams are used to decompose an association between variables into different types of relationships^14^. In a path analysis model, in addition to the direct effect, there is also an indirect effect of independent variables, via a mediating variable, on the outcome variable. In path diagrams, ellipses designate hypothetical factors, and squares designate manifest covariates. Likewise, single-headed arrows designate the impact of one covariate on another while double-headed arrows designate relationships among pairs of covariates.

However, SEMs are not equipped to handle nonlinear relationships. To resolve this issue, generalized structural equation models (GSEM) were developed to combine the power and flexibility of the structural equation model in a unified modeling framework to handle endogenous observable categorical responses^15^. The classic SEM could be moved towards a more general nonlinear or nonparametric form by writing the latent variable model as

1 $${\eta }_{i}={f}_{\eta }\left({\eta }_{i},{\xi }_{i},{\zeta }_{i}\right)$$

The two-equation measurement models are

2 $$\begin{aligned}&{{\varvec{y}}}_{i}={f}_{y}\left({{\varvec{\eta}}}_{{\varvec{i}}},{\varepsilon }_{i}\right)\\ &{{\varvec{x}}}_{i}={f}_{x}\left({{\varvec{\xi}}}_{{\varvec{i}}},{\delta }_{i}\right)\end{aligned}$$

where ***x*** and ***y*** are vectors of observable variables, ${{\varvec{\eta}}}_{{\varvec{i}}}$ are the latent endogenous variables, ${{\varvec{\xi}}}_{{\varvec{i}}}$ are the latent exogenous variables, ${\varepsilon }_{i}$ is the vector of unique factors (or disturbances) that consists of all the other influences on ${{\varvec{y}}}_{i}$ that are not part of ${{\varvec{\eta}}}_{{\varvec{i}}}$ and ${\delta }_{i}$ is a random vector of error measurements or residuals. The functions (${f}_{\eta }$ ${f}_{x}$,${f}_{y}$) provide a general way to represent the connections between the variables within the parentheses to those on the left-hand side of each equation.

### Parameter estimation

Generalized structural equation models fit generalized linear models with latent variables via maximum likelihood. The family/link combinations that the generalized structural equation models allow are Bernoulli (logit, probit, and cloglog) and Binomial (logit, probit, and cloglog). For $q$-point Gauss–Hermite quadrature (GHQ), let the abscissa and weight pairs be denoted by (${{a}_{k},}^{*},{{\omega }_{k}}^{*}$)$,k=\mathrm{1,2},\dots q$. Then GHQ approximation is the,

3 $$\underset{-\infty }{\overset{\infty }{\int }}f(x)\mathrm{exp}(-{x}^{2}){d}_{x}\approx \sum_{k=1}^{q}{{w}_{k}}^{*}f({{a}_{k}}^{*})$$

Using the standard normal distribution yields the approximation

4 $$\underset{-\infty }{\overset{\infty }{\int }}f(x)\phi (x){d}_{x}\approx \sum_{k=1}^{q}{w}_{k}f({a}_{k})$$

where $${a}_{k}=\sqrt{2{{a}_{k}}^{*}}$$ and $$ w_{k}  = {\raise0.7ex\hbox{${(w_{k} ^{*} )}$} \!\mathord{\left/ {\vphantom {{(w_{k} ^{*} )} {\sqrt \pi  }}}\right.\kern-\nulldelimiterspace} \!\lower0.7ex\hbox{${\sqrt \pi  }$}} $$

Let ${\varvec{v}}$ be a random vector whose elements are independent standard normal, and let ${\varvec{L}}$ be the Cholesky decomposition of ${{\varvec{\Sigma}}}_{\upsilon }$, that is, ${{\varvec{\Sigma}}}_{\upsilon } = {\varvec{L}}{{\varvec{L}}}^{^{\prime}}$. In the distribution, we have that ${\varvec{u}} = {\mu }_{\upsilon }+ {l}_{\upsilon }$, and the linear predictions vector as a function of $\upsilon$ is

5 $${z}_{ij}={{\varvec{x}}}_{j}^{^{\prime}}{{\varvec{\beta}}}_{{\varvec{i}}}+{x}_{j}^{^{\prime}}{{\varvec{\Lambda}}}_{{\varvec{i}}}\left({{\varvec{\mu}}}_{{\varvec{v}}},\boldsymbol{ }+{{\varvec{L}}}_{{\varvec{\upsilon}}}\right)$$

Therefore, the likelihood of a given cluster is

6 $$ {\mathcal{L}}\left( \theta  \right) = (2\pi )^{{ - {\raise0.7ex\hbox{$r$} \!\mathord{\left/ {\vphantom {r 2}}\right.\kern-\nulldelimiterspace} \!\lower0.7ex\hbox{$2$}}}} \mathop {\mathop \smallint \limits^{\infty } }\limits_{{ - \infty }}  \ldots \mathop {\mathop \smallint \limits^{\infty } }\limits_{{ - \infty }} exp\left\{ {logf\left( {y,z,\theta } \right) - {\raise0.7ex\hbox{$1$} \!\mathord{\left/ {\vphantom {1 2}}\right.\kern-\nulldelimiterspace} \!\lower0.7ex\hbox{$2$}}\sum {\upsilon _{k} ^{2} } } \right\}d_{{\upsilon _{1} }}  \ldots d_{{\upsilon _{r} }}  $$

where $r$ is the number of latent variables. Consider an r-dimensional quadrature grid containing q quadrature points in each dimension. Let the vector of abscissas ${{\varvec{a}}}_{k}={\left({a}_{{k}_{1}} , . . . , {a}_{{k}_{r}} \right)}^{^{\prime}}$ be a point in this grid and let ${{\varvec{\omega}}}_{k}={\left({\omega }_{{k}_{1}} , . . . , {\omega }_{{k}_{r}}\right)}^{^{\prime}}$ be the vector of corresponding weights. The likelihood is then approximated with

7 $${\mathcal{L}}^{{\varvec{G}}{\varvec{H}}{\varvec{Q}}}\left({\varvec{\theta}}\right)=\sum_{{{\varvec{k}}}_{1}=1}^{{\varvec{q}}}\dots \sum_{{{\varvec{k}}}_{{\varvec{r}}}=1}^{q}\left[exp\left\{\sum_{i=1}^{n}\mathrm{log}{f}_{i}\left({y}_{ij},{{\varvec{z}}}_{ijk},{\varvec{\theta}} \right)\right\}\prod_{s=1}^{r}{{\varvec{\omega}}}_{{k}_{s}}\right]$$

where

$${z}_{ijk}={x}_{j}^{^{\prime}}{\varvec{\beta}}+{x}_{j}^{^{\prime}}{{\varvec{\Lambda}}}_{{\varvec{i}}}\left({{\varvec{\mu}}}_{{\varvec{v}}},\boldsymbol{ }+{\varvec{L}}{\boldsymbol{\alpha }}_{{\varvec{k}}}\right)$$

Again, let us reconsider the likelihood in (7). If we fix the observed variables and the model parameters, we see that the posterior density for ${\varvec{v}}$ is proportional to

8 $$\phi (\upsilon )f({\varvec{y}},{\varvec{z}},{\varvec{\theta}})$$

It is reasonable to assume that this posterior density can be approximated by a multivariate normal density with mean vector $\mu v$ and variance matrix ${\varvec{\tau}}{\varvec{v}}$. Instead of using the prior density of $v$ as the weighting distribution in the integral, we can use our approximation for the posterior density,

9 $$\mathcal{L}\left(\theta \right)={\int }_{{\mathfrak{R}}^{{\varvec{r}}}}^{{\varvec{n}}}\frac{f\left({\varvec{y}},{\varvec{z}},{\varvec{\theta}}\right)\phi \left({\varvec{\upsilon}}\right)}{\phi \left({\varvec{\upsilon}},{{\varvec{\mu}}}_{{\varvec{\upsilon}}},{{\varvec{\tau}}}_{{\varvec{\upsilon}}}\right)}\phi ({\varvec{\upsilon}},{{\varvec{\mu}}}_{{\varvec{\upsilon}}},{{\varvec{\tau}}}_{{\varvec{\upsilon}}}){d}_{{\varvec{\upsilon}}}$$

The likelihood is then approximated with

10 $${\mathcal{L}}^{\mathbf{*}}\left({\varvec{\theta}}\right)=\sum_{{{\varvec{k}}}_{1}=1}^{{\varvec{q}}}\dots \sum_{{{\varvec{k}}}_{{\varvec{r}}}=1}^{q}\left[exp\left\{\sum_{i=1}^{n}\mathrm{log}{f}_{i}\left({y}_{ij},{{\varvec{z}}}_{ijk}^{*} ,{\varvec{\theta}} \right)\right\}\prod_{s=1}^{r}{{\varvec{\omega}}}_{{k}_{s}}\right]$$

where

$${z}_{ijk}^{*}={x}_{j}^{^{\prime}}{\varvec{\beta}}+{x}_{j}^{^{\prime}}{{\varvec{\Lambda}}}_{{\varvec{i}}}\left({{\varvec{\mu}}}_{{\varvec{v}}},\boldsymbol{ }+{\varvec{L}}{\boldsymbol{\alpha }}_{{\varvec{k}}}\right)$$

and ${\boldsymbol{\alpha }}_{{\varvec{k}}}$ and the ${\omega }_{{k}_{s}}$ are the adaptive versions of the abscissas and weights after an orthogonalyzing transformation and are functions of ${{\varvec{a}}}_{{\varvec{k}}}$ and ${{\varvec{\omega}}}_{k}$. The adaptive parameters ${{\varvec{\mu}}}_{v}$ is the posterior mode for **v** and ${{\varvec{\tau}}}_{v}$ is the curvature at the mode.

## Results

### Descriptive results

Table 1 shows the percentage distribution of childhood malnutrition and morbidity. This data analysis comprised 8742 samples of under-five children. The prevalence of malnutrition and morbidity was more common among children exposed to anemia in comparison to non-anemic children. Anemic children had a cough (16.7%), diarrhea (11.8%), fever (14.5), stunted (36.4%), wasted (12.1%), and underweight (24.9%). It is more common for children born to mothers with no formal education to suffer from stunting (40.3%), wasting (13.7%), and underweight (29.30%) than children born to mothers with formal education. Children born to a malnourished mother are more affected by malnutrition indicators; stunting (34.9%), wasting (10.4%) and underweight (21.9%) compared to children born to a nourished mother. Furthermore, children from households who do not have access to improved toilet facilities were more at risk of stunting (40.4%), wasting (15.2%), and being underweight (30.4%) compared to those who use toilet facilities like flush and latrine facilities. Children born at home are more at risk of stunting (40.2%), wasting (13.6%), and being underweight (29.1%) compared to children delivered at health centers. Children from poor households were more exposed to malnutrition indicators, stunting (41.6%), wasting (14.9%), and underweight (31.5%) compared to children from middle- and high-income households.

**Table 1:** Percentage distribution of childhood malnutrition and morbidity.

Risk factors	Had cough Percent	Had diarrhea Percent	Had fever Percent	Stunted Percent	Wasted Percent	Underweight Percent
**Mother educational level**						
No education	16.0	11.1	13.6	40.3	13.7	29.5
Primary	18.9	12.9	16.3	33.8	10.0	19.5
Secondary and higher	14.7	11.8	14.3	19.0	7.6	10.4
**Source of drinking water**						
Pied	17.9	12.1	15.9	22.4	6.6	11.5
Public tap	16.4	11.0	14.0	38.7	13.0	27.4
Protected spring	17.1	13.0	14.7	39.2	11.6	26.7
Other	16.1	12.0	14.2	38.3	13.3	26.7
**Toilet facility**						
Flush toilet	20.0	10.3	15.1	18.3	8.0	10.3
Latrine	15.6	11.7	13.8	34.2	9.6	21.1
No facility	17.4	11.7	15.0	40.4	15.2	30.4
Wealth index: Poor	15.9	11.1	14.0	41.6	14.9	31.5
Middle	17.0	12.5	13.9	37.5	10.9	24.2
Rich	17.5	2.1	15.2	27.3	8.0	14.2
**Breastfeeding**						
Not breastfed	16.6	10.3	14.4	38.2	10.4	25.4
Breastfed	16.6	12.3	14.3	35.5	12.9	24.6
Anemia level: Anemic	16.7	11.8	14.5	36.4	12.1	24.9
Not anemic	12.6	6.3	9.0	35.6	10.4	24.3
**Husband’s education level**						
No education	15.5	10.3	13.3	40.8	13.8	30.1
Primary	19.9	13.3	16.6	35.3	9.9	21.5
Secondary	15.1	13.9	15.2	28.3	12.1	17.7
Higher	11.3	11.3	11.1	19.8	9.1	11.9
**Mother work status**						
Not work	15.8	11.1	13.6	36.1	12.6	25.3
Had work	18.5	12.9	16.4	37.0	10.8	23.7
Birth order number						
1st order	18.7	12.3	15.5	33.0	10.7	20.5
2–3rd order	16.0	11.6	13.8	33.9	10.7	22.6
4th and above order	16.1	11.4	14.3	39.4	13.6	28.2
Sex of child: Male	16.6	12.2	14.5	38.0	13.2	26.0
Female	16.5	11.1	14.2	34.6	10.9	23.7
**Preceding birth interval**						
Less than 24 months	16.7	11.6	14.4	37.2	12.6	26.1
24–47 months	16.1	10.9	13.7	37.7	13.1	27.3
48 and above months	17.2	13.2	15.7	31.8	8.8	17.6
**Age of the child**						
0–11 months	17.9	13.9	16.0	13.1	16.3	13.3
12–23 months	19.8	17.9	19.5	38.5	14.8	25.3
24–59 months	15.0	8.6	12.0	44.1	9.6	29.0
Place of delivery: home	15.6	10.7	13.3	40.2	13.6	29.1
Health center	18.4	13.4	16.4	29.4	9.2	17.2
**Mother's BMI**						
≥ Equal to18.5	16.9	11.5	15.3	41.0	17.3	34.1
Less than 18.5	16.5	11.7	14.1	34.9	10.4	21.9

### Results from generalized structural equation models

The data analysis was done using SPSS AMOS version 25.0 and R 3.6.2 package gsem. To select the appropriate link functions of malnutrition and morbidity indicators for GSEM, we first selected the Bernoulli distributions available. Only three link functions, logit, probit, and complementary log–log, were supported. We intended to identify an appropriate link function that guarantees the goodness-of-fit of the binomial regression models under symmetric or asymmetric to identify risk factors of child malnutrition and morbidity. The motivation behind a model selection is to compare the relative value of link functions and determine which one is the best fit for the observed data. The AIC and BIC are the most common methods of model selection. Then, we employed the GSEM model using Bernoulli distribution with logit, probit, and complementary log–log link functions. Model comparison was made using AIC and BIC to select the best-fit model. The model with the logit link function is selected as the best-fit model for the data (Table 2). The direct, indirect, and total effects were estimated in a logit model. The indirect effects are obtained by multiplying the slope coefficients on each path and the total effects are the sum of direct and indirect effects. For example, we can calculate the indirect and total effects for the following path diagram as follows:
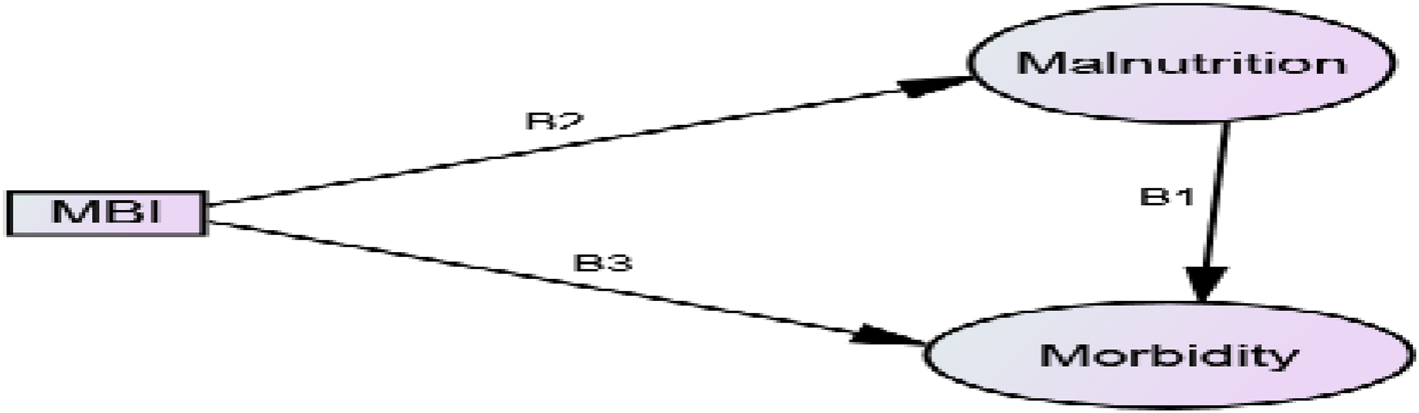

$$indirect \,effect={\beta }_{1}{\beta }_{2}$$

$$total \,effect={\beta }_{3}+{\beta }_{1}{\beta }_{2}$$

where ${\beta }_{1}$= path coefficient for morbidity $\leftarrow$ malnutrition, ${\beta }_{2}$= path coefficient for malnutrition $\leftarrow$ mother body mass index (MBI), ${\beta }_{3}$= path coefficient for morbidity $\leftarrow$ mother body mass index (MBI).

**Table 2:** Model comparison under different link functions.

Family	Link	LL	AIC	BIC
Bernoulli	**Logit**	**− 20,479.64**	**41,093.28**	**41,565.19**
	Probit	− 20,543.88	41,221.76	41,693.67
	cloglog	− 21,064.1	42,262.2	42,734.12

The covariates sex of the child and age of the child included in the model as direct with the indicators of malnutrition and morbidity. This implies that the observed causes sex of the child and age of the child determine the observed indicators of stunting, wasting, underweight, diarrhea, cough, and fever. Furthermore, the finding obtained from the GSEM revealed a significant association between latent factors, morbidity, and malnutrition.

#### Direct effects

Table 3 shows the estimated coefficients of the direct effects of selected risk factors of child malnutrition and morbidity outcomes from the fitted generalized structural equation model. The results revealed that the child’s anemia status, preceding birth interval, breastfeeding, mother’s nutritional status, education level, work status, places of delivery, and household wealth index had a direct effect on the child's nutritional status. The child’s nutritional status, birth order number, anemia status, breastfeeding; mother’s nutritional status, work status; household’s source of drinking water, types of toilet facility, and husband's education level had a direct effect on the child's health status. Our result indicates that the probability of a child being malnourished is significantly lower among children born to nourished mothers (AOR = 0.71, 95% CI 0.68–0.75). The result suggests that malnourished children are significantly more exposed to morbidity (AOR = 1.14, 95% CI 1.09–1.19). Similarly, a child born to a nourished mother is less exposed to morbidity (AOR = 0.92, 95% CI 0.85–1.00). A child born with short birth spacing is more likely affected by malnutrition. It resulted that children born with enough birth space between 24 and 47 months and 48 months and above are less affected by malnutrition (AOR = 0.93, 95% CI 0.88–0.99 and AOR = 0.71, 95% CI 0.65–0.76), respectively. Our analysis indicated that birth order significantly affects child health. A child born second to third and fourth and above order (AOR = 0.87, 95% CI 0.77–0.99 and AOR = 0.88, 95% CI 0.79–0.99) are less likely diseased than first birth order, respectively. The analysis indicates that being from middle and rich households significantly lowers the risk of malnutrition (AOR = 0.85, 95% CI 0.78–0.91 and AOR = 0.66, 95% CI 0.61–0.72), respectively. From Table 3, it is indicated that the level of education of mothers significantly affects the nutritional status of the child, where malnutrition decreases as the level of education of mother’s increases.

**Table 3:** Direct effects of risk factors on child malnutrition and morbidity from GSEM.

Covariates	Malnutrition AOR (95% CI)	Morbidity AOR (95% CI)
Malnutrition	–	1.14** (1.09–1.19)
MBI (ref: < 18.5) $\ge$ 18.5	0.71** (0.68–0.75)	0.92* (0.85–1.00)
**Birth order (1st)**		
2nd–3rd order	0.95 (0.85–1.07)	0.87* (0.77–0.99)
4th and above	1.02 (0.93–1.12)	0.88* (0.79–0.99)
**Birth interval (ref: < 24 months)**		
24–47 months	0.93* (0.88–0.99)	0.92 (0.83–1.02)
48 and above month	0.71*** (0.65–0.76)	0.95 (0.85–1.07)
**Wealth index( ref: poor)**		–
Middle	0.85** (0.78–0.91)	–
Rich	0.66** (0.61–0.72)	–
**Mother education (ref: no education)**		–
Primary	0.79** (0.75–0.85)	–
Secondary and higher	0.57** (0.41–0.78)	–
**Source of drinking water (ref: piped)**		
Other	0.89 (0.78–1.03)	0.83* (0.71–0.98)
Protected spring	1.07 (0.92–1.24)	0.83* (0.69–1.01)
Public tap	0.98 (0.87–1.12)	0.85* (0.73–0.99)
**Toilet facility (ref: No toilet facility)**		
Latrine	0.95 (0.79–1.15)	0.88* (0.75–1.03)
Flush	0.91 (0.75–1.11)	0.79** (0.67–0.92)
Breastfeed(ref: No) yes	0.90** (0.85–0.96)	0.76** (0.69–0.82)
Anemia : (Ref: Anemic)Not anemic	0.81* (0.68–0.96)	0.75* (0.54–1.03)
Mothe work status: yes	1.08* (1.02–1.14)	1.20** (1.11–1.29)
Pdelivery :health	0.81** (0.75–0.88)	1.04 (0.95–1.13)
**Husband/partner education (Ref: No educ)**		
Primary	–	1.26 (1.06–1.25)
Secondary	–	1.01 (0.88–1.15)
Higher	–	0.76** (0.64–0.89)

*AOR* Adjusted Odds Ratio, *CI* confidence interval.

**P < 0.001; *P < 0.05.

Furthermore, it is revealed that a mother with primary or secondary and higher education levels is (AOR = 0.79, 95% CI 0.75–0.85 and AOR = 0.57, 95% CI 0.41–0.78), respectively, times less likely to be malnourished as compared to a mother with no formal education. Children from families using public tap water, protected spring water and other sources of water are less likely to be diseased compared to households that use piped water, which is an unexpected result. Moreover, children from a household that uses a latrine and flush toilet facility are less likely diseased compared to families that have no toilet facility. The analysis indicated that the breastfeeding status of children significantly affects the nutritional and morbidity status of children in Ethiopia. Children being breastfed are (AOR = 0.90, 95% CI 0.85–0.96 and AOR = 0.79, 95% CI 0.67–0.92), respectively, times less likely to be malnourished and diseased as compared to non-breastfed children. Similarly, children not anemic are (AOR = 0.81, 95% CI 0.68–0.96) times less likely to be malnourished and diseased as compared to non-breastfed children. The work status of the mother plays a critical role in fixing the nutritional and health status of her child. Children from working mothers are (AOR = 1.08, 95% CI 1.02–1.14 and AOR = 1.20, 95% CI 1.11–1.29), respectively, times more likely to be malnourished and diseased as compared to children from not working mothers. Our analysis indicates that the probability of a child being diseased is significantly lower for children from a husband-educated higher level (AOR = 0.76, 95% CI 0.64–0.89) compared to uneducated husbands.

#### Indirect and total effects

Table 4 shows the indirect and total effects of risk factors on child morbidity via malnutrition. The estimation results for parametric indirect effects indicated that breastfeeding, mother nutritional status, child anemic status, place of delivery, household wealth index, and childbirth spacing exhibit an indirect effect on morbidity through malnutrition. For instance, the result revealed that children who breastfed (AOR = 0.98, 95% CI 0.80–0.99) were times less likely to be exposed to diseases than non-breastfed children via malnutrition. Children from nourished mothers are (AOR = 0.96, 95% CI 0.94–0.097) times less likely to be diseased. Birth order, source of drinking water, and types of toilet facilities have a significant total effect on morbidity through malnutrition, but they have an insignificant indirect effect on morbidity. Furthermore, the result shows that children from middle and high-income households are (AOR = 0.97, 95% CI 0.96–0.99 and AOR = 0.94, 95% CI 0.93–0.96), respectively, times less likely to be exposed to morbidity than children from poor families through malnutrition. likewise, the analysis indicated that with birth spacing 24–47 months and 48 months and above are (AOR = 0.99, 95% CI 0.98–1.00 and AOR = 0.96, 95% CI 0.94–0.97), respectively, times less likely to be affected by morbidity than children with short birth space via malnutrition, but they have an insignificant direct effect on morbidity.

**Table 4:** Indirect and total effects of risk factors on child morbidity via malnutrition.

Paths via malnutrition	Indirect effect AOR (95% CI)	Total effect AOR (95% CI)
Breast $\to$ Morbidity	0.98 (0.80–0.99)*	0.74 (0.69–0.81)
MBI $\to$ Morbidity	0.96 (0.94–0.97)**	0.88 (0.81–0.95)*
2nd–3rd order $\to$ Morbidity	0.99 (1.01–1.00)	0.87 (0.77–0.98)*
4th and above $\to$ Morbidity	1.00 (0.99–1.01)	0.88 (0.79–0.99)*
Not anemic $\to$ Morbidity	0.38 (2.58–2.69)*	0.72 (0.52–1.01)*
Mothe work status: yes $\to$ Morbidity	1.00 (1.00–1.02)*	1.21 (1.12–1.31)**
Other $\to$ Morbidity	0.98 (0.96–1.00)	0.82 (0.70–0.96)*
Protected spring $\to$ Morbidity	1.01 (0.99–1.03)	0.84 (0.69–1.01)
Public tap $\to$ Morbidity	0.99 (0.98–1.01)	0.85 (0.73–0.98)*
No toilet facility $\to$ Morbidity	0.99 (0.97–1.02)	0.87 (0.74–1.02)
Latrine $\to$ Morbidity	0.99 (0.96–1.01)	0.78 (0.67–0.91)*
Pdelivery : health center $\to$ Morbidity	0.98 (0.96–0.98)**	1.00 (0.92–1.09)*
Wealth Index: Middle $\to$ Morbidity	0.97 (0.96–0.99)**	0.95 (0.86–1.05)*
Wealth Index: Rich $\to$ Morbidity	0.94 (0.93–0.96)**	0.96 (0.94–0.98)**
PBI: 24–47 months $\to$ Morbidity	0.99 (0.98–1.00)*	0.91 (0.82–1.00)*
PBI: 48 and > months $\to$ Morbidity	0.96 (0.94–0.97)**	0.91 (0.81–1.03)*

*AOR* Adjusted Odds Ratio, *CI* confidence interval.

**P < 0.001; *P < 0.05.

**Figure 2:**
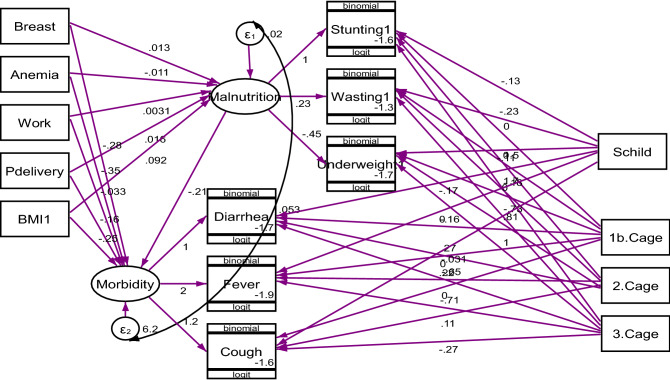
Generalized structural equation model of effects of selected covariates on latent variables malnutrition and morbidity and observed indicators.

From Fig. 2, the sex of the child and age of the child significantly determine the stunting, wasting, underweight, diarrhea, and fever status of under-5 children. Being a female child is (AOR = 0.88. 95% CI 0.79–0.98 and AOR = 0.89, 95% CI 0.69–0.93), respectively, times less likely to be stunted and wasted than the male child. Children aged 12–23 months and 24–59 months are (AOR = 4.29, 95% CI 3.53–5.22 and AOR = 5.58, 95% CI 4.62–6.68), respectively, times more likely exposed to growth retardation than younger (Fig. 2). Similarly, a child aged 12–23 months and 24–59 months are (AOR = 2.25, 95% CI 1.81–2.80 and AOR = 2.71, 95% CI 2.25–3.25) respectively, times more likely exposed to underweight than younger. However, a child aged 24–59 months is 0.48 (95% CI 0.40–0.55) times less likely to be affected by wasting than the male counterpart (Fig. 2). As can be observed from Fig. 2, a child aged between 24 and 59 months is (AOR = 0.52, 95% CI 0.39–0.69 and AOR = 0.48, 95% CI 0.28–0.82), respectively, times less likely to have diarrhea and fever compared to the younger.

## Discussion

We have used a generalized structural equation model to fit the morbidity (diarrhea, fever, and cough) and malnutrition (stunting, wasting, and underweight) using the 2016 Ethiopia Demographic and Health survey data. The generalized structural equation models offer latent effects on child malnutrition and morbidity within a combined modeling framework. Besides, generalized structural equation modeling permitted analysis of malnutrition as a mediator of the association between selected risk factors and latent variable morbidity.

The analysis indicated that the mother’s nutritional status, education level, breastfeeding, work status and places of delivery; child’s birth space and anemia status, and household’s economic status had a direct effect on the child's nutritional status. The child’s nutritional status, birth order, anemia status**;** mother’s nutritional status, breastfeeding and work status; household’s source of drinking water, toilet facility, and husband's education level had a direct effect on the child's health status. Furthermore, the mother’s nutritional status, breastfeeding and place of delivery; the child's anemic status and birth spacing, and the household’s economic status had an indirect effect on morbidity through malnutrition.

The result indicates that the probability of a child being malnourished is significantly lower among children born to nourished mothers. Similar to this finding^2,16–18^ confirmed that a mother’s nutritional status can affect her ability to give the necessary care to her children. This research indicated that the level of education of mothers significantly affects the nutritional status of the child, where malnutrition decreases as the level of education of mother’s increases. This finding is in line with other previous studies^2,19^. This shows that an educated mother is more likely to learn proper feeding practices, improve hygiene, and gain improved access to knowledge and awareness. An important factor affecting a child's nutritional and health status is the working status of the mother. Children from working mothers are more likely to be malnourished and diseased as compared to children from not working mothers. The finding is consistent with some previous studies^3,7,16^. Children being breastfed are less likely to be malnourished and diseased as compared to non-breastfed children. This result is consistent with previous findings in^5,18,20^. Children not anemic are less likely to be malnourished and diseased as compared to anemic children. Some studies found similar results^2,21^. A child born with short birth spacing is more likely to suffer from malnutrition. Female children are less likely to be stunted and wasted than male children. Growth retardation is more common in children who are 12–23 months of age and 24–59 months of age. Similarly, A child aged 12–23 months and 24–59 months is more likely to suffer from being underweight than a younger child. A child aged 24–59 months is, however, less likely to be affected by wasting than a male child of the same age. When compared with younger children, older children are at lower risk for diarrhea and fever. The results are consistent with^2,7,18^. In comparison to the first birth order, children born second to third and fourth and above are less likely to suffer from the disease. Similar results are shown in^3,20^. Children from families who use public water, protected springs, or other sources of water are less likely to be sick than children from households who use piped water, an unexpected finding. Contradictory findings have been found by some researchers^21,22^. Children from a household that uses a latrine and flush toilet facility are less likely to be ill compared to those from families without toilet facilities. This finding confirms with^3,16^. According to our analysis, children of husbands with higher levels of education are significantly less likely to become ill than those whose husbands are uneducated. The result revealed that children who breastfed were less likely to be exposed to diseases than non-breastfed children via malnutrition. The chances of a child being sick are lower when their mother is well-fed. Birth order, source of drinking water, and types of toilet facilities have a significant total effect on morbidity through malnutrition, but they have an insignificant indirect effect on morbidity. Also, children from middle- and high-income households are less likely to suffer from malnutrition-related morbidity than children from poor families. According to the analysis, children with a birth spacing of 24–47 months and 48 months and above are less likely to be affected by morbidity than children with short birth spacing via malnutrition, although their direct effect on morbidity is insignificant.

## Conclusion

This research addresses the common predictors of malnutrition and morbidity in children under five using generalized structural equation models. Child malnutrition and morbidity were considered latent variables. This investigation has indicated that childhood malnutrition and morbidity in Ethiopia is still a key health problem that needs to be urgently addressed.

Using a generalized structural equation model with logit link function: the mother’s nutritional status, education level, breastfeeding, work status, and places of delivery; the child’s birth space and anemia status, and the household’s economic status had a direct effect on the child's nutritional status. The child’s nutritional status, birth order, anemia status; mother’s nutritional status, breastfeeding and work status; household’s source of drinking water, toilet facility, and husband's education level had a direct effect on the child's health status. Furthermore, the mother’s nutritional status, breastfeeding and place of delivery; the child's anemic status and birth spacing, and the household’s economic status had an indirect effect on morbidity through malnutrition. This study concludes that child malnutrition and morbidity are influenced by no single factor, hence dealing with child malnutrition and morbidity will need comprehensive plans.

### Limitation of the study

Demographic and Health Survey data are cross-sectional. Consequently, it is not supported for understanding the changes in malnutrition and morbidity over time. Accordingly, future studies should consider many DHS datasets as a mimic of a longitudinal study to examine the stability of the significant risk factors obtained in this study over time.

## Data Availability

The data can be accessed from www.dhsprogram.com/

## Abbreviations

AOR: Adjusted Odds Ratio
AIC: Akaike Information Criteria
BIC: Bayesian Information Criteria
CI: Confidence Interval
DHS: Demographic and Health Survey
GSEM: Generalized Structural Equation Models
MBI: Mother body mass index
MIMIC: Multiple Indicators and Multiple Causes
SEM: Structural equation modeling
